# Initial Endoscopic Ventricular Failure to Relieve Hydrocephalus in Patients With Obstructing Brain Tumors Predicts a Histone 3K27M Mutation

**DOI:** 10.7759/cureus.79362

**Published:** 2025-02-20

**Authors:** Lisa B Shields, Michael W Daniels, Lennea Coombs, Alexandra Vaynerman, Kaylyn Sinicrope, Mustafa Barbour, Aaron Spalding, William Gump, Ian Mutchnick, David Sun

**Affiliations:** 1 Norton Neuroscience Institute, Norton Healthcare, Louisville, USA; 2 Department of Bioinformatics and Biostatistics, University of Louisville, Louisville, USA; 3 Norton Cancer Institute, Norton Healthcare, Louisville, USA

**Keywords:** endoscopic third ventriculostomy, h3k27m mutation, hydrocephalus, midline gliomas, neurosurgery, radiation oncology, ventriculoperitoneal shunt

## Abstract

Background

Diffuse midline gliomas (DMG) are a subset of malignant gliomas that could be linked to an H3K27M mutation. Hydrocephalus may be the initial presenting condition because of its frequent pontine location. This study evaluated the outcomes of tumor resection, endoscopic third ventriculostomy (ETV), and ventriculoperitoneal shunt (VPS) placement in DMG patients compared to wild-type (WT) tumors in treating hydrocephalus.

Materials and methods

We identified newly diagnosed pediatric and adult patients with midline tumors over an eight-year period (September 14, 2016-July 1, 2024).

Results

Out of a total of 74 patients, 20 (27.0%) patients were diagnosed with an H3K27M mutation, and 24 (32.4%) presented with hydrocephalus. Patients with a DMG H3K27M mutant (15 patients) were statistically more likely to be diagnosed with hydrocephalus compared to those with a WT midline glioma (9 patients) (p<0.001). Of the 24 patients with hydrocephalus, 8 (34.8%) underwent a VPS placement, 5 (22.7%) had tumor resection, 5 (21.7%) underwent an ETV, and 4 (17.4%) had both a VPS and ETV. A significant difference in hydrocephalus-free survival was observed among the treatment groups (p=0.0013). ETV failure was significantly higher in H3K27M patients while VPS was more successful in managing hydrocephalus.

Conclusions

As H3K27M mutation analysis is not available rapidly when patients initially present with midline gliomas, neurosurgeons use their best clinical judgment regarding the management of hydrocephalus. VPS demonstrated superior outcomes compared to ETV in controlling hydrocephalus among patients with a DMG H3K27M mutation in the present study; therefore, neurosurgical teams should have increased vigilance following ETV in this population.

## Introduction

Diffuse midline gliomas (DMGs) are a rare subtype of glial tumor with extensive morphologies (gangliogliomas, anaplastic gangliogliomas, pilocytic astrocytomas, and posterior fossa ependymomas [1-3]. A DMG with an H3K27M mutation substitutes an amino acid on histone H3 that promotes gene expression and tumor growth [4]. This recurrent somatic gain-of-function mutation results from lysine to methionine (p.Lys27Met: K27M) substitution at codon 27 in histone 3 (H3) variants (H3 isoforms H3.3 gene and H3.1 gene), which results in altered DNA methylation and gene expression profiles that spur gliomagenesis [1,4-8]. In 2016, the World Health Organization (WHO) classified “a diffuse midline glioma, H3K27M-mutant” as a new distinct entity that is designated grade IV even when mitotic figures, microvascular proliferation, and necrosis are not observed [9]. This particular tumor mutation usually has a worse prognosis than wild-type (WT) midline gliomas, especially in the pediatric population [4,10]. However, it has been shown to have a longer median survival compared with high-grade WT tumors in adults [7,11].

Studies have compared the incidence, sites of origin, prognosis, and molecular pathogenesis of the DMG H3K27M mutant in children versus adults and have highlighted the heterogeneous features [3,11,12]. This tumor mutation is the second most common but most fatal childhood malignant brain tumor, with 200-400 cases annually in the United States [4,13]. Initially described in pediatric diffuse intrinsic pontine glioma (DIPG), this mutation has an aggressive clinical behavior with a poor prognosis in children [3-5,10]. The median overall survival is 9-15 months in children versus 9.3-27.6 months in adults [3,4,6,7,12-15]. The pons is the most frequent site for children compared to the thalamus and spinal cord in adults [4,7,12,13,15,16]. This incidence of the H3K27M mutation in DMG is also higher in children (80%) versus adults (15-60%) [15]. In studies focusing on adults with the DMG H3K27 mutation, the prognosis is not as poor as that seen in children, prompting investigators to question the grade IV diagnosis of all DMGs [17]. Better survival in adults is associated with a low histological grade, KPS ≥ 80, age ≤ 60 years, and gross total resection (GTR) of the tumor [17].

While common clinical symptoms of the DMG H3K27 mutation include dysphagia, slurred speech, and long-tract signs, hydrocephalus may be the initial presenting condition [4,10]. Few studies have addressed the treatment of hydrocephalus in patients with a DMG H3K27 mutation [1,18-24].

We report 74 patients with newly diagnosed midline gliomas, 20 of whom had an H3K27 mutation and 24 of whom developed concurrent hydrocephalus and were treated with tumor resection or a CSF diversion procedure (ventriculoperitoneal shunt (VPS) and/or endoscopic third ventriculostomy (ETV)). The main scope of this work is to highlight the importance of patients who turned out to have an underlying H3K27M mutation and are at high risk for failure from ETV. The clinical characteristics and survival analysis of these patients are highlighted. The benefit of using either an ETV or a VPS in patients with a DMG H3K27 mutation and hydrocephalus is also discussed.

## Materials and methods

**Figure 1:**
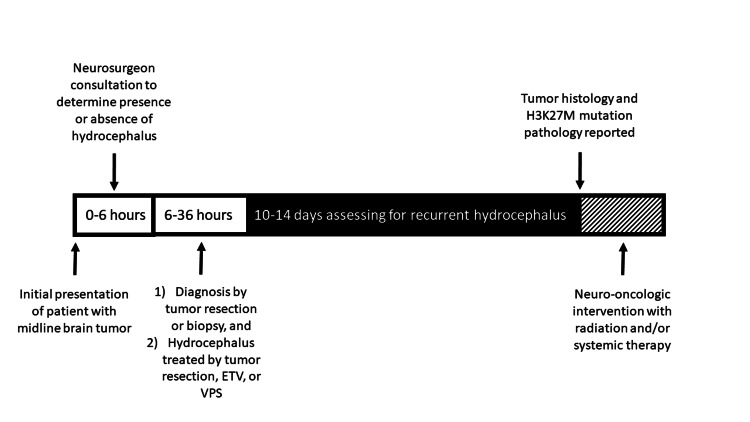
Timeline of management of a patient who presents with a midline brain tumor ETV: endoscopic third ventriculostomy; VPS: ventriculoperitoneal shunt

**Figure 2:**
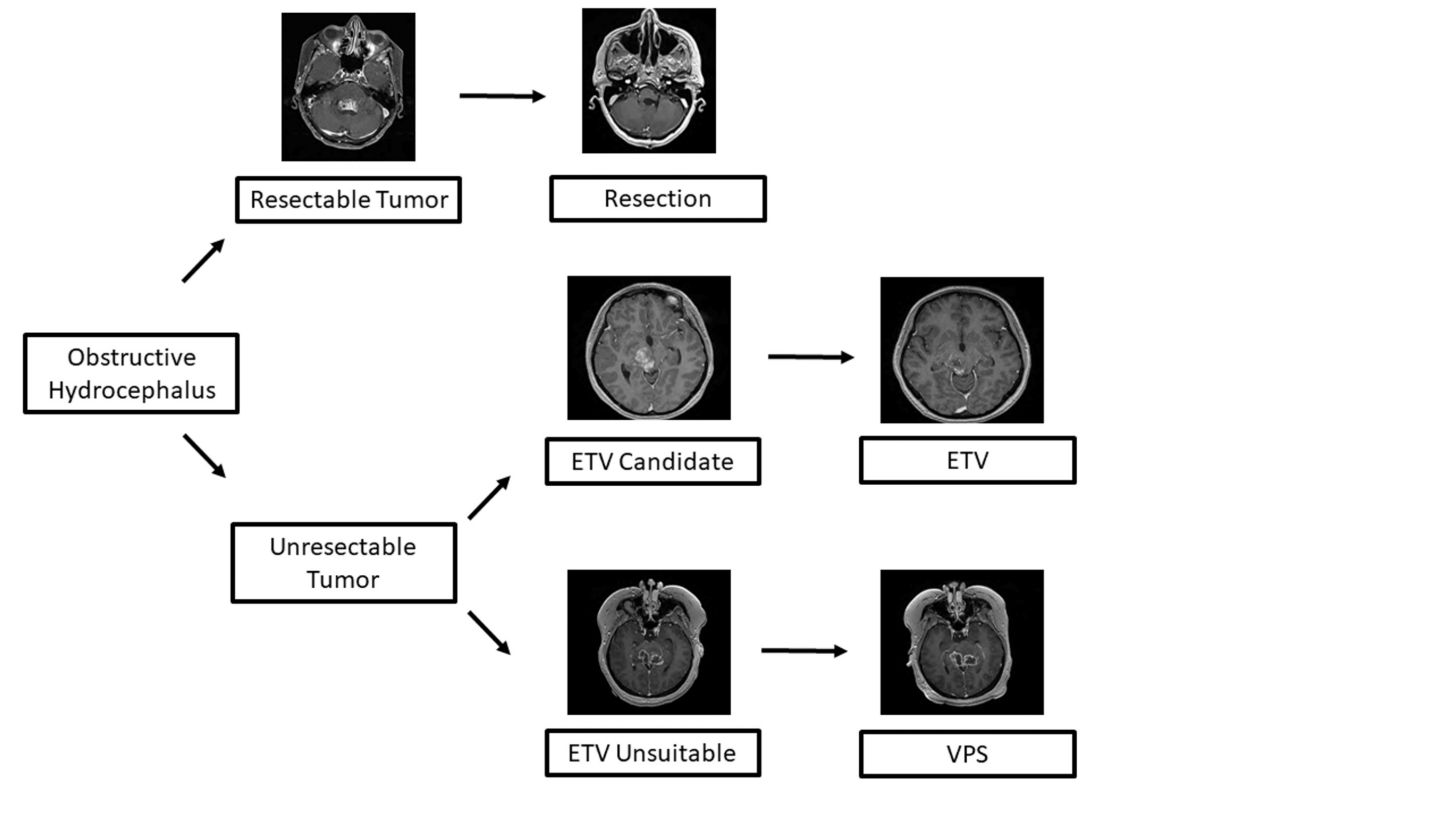
Flowchart for the treatment of obstructive hydrocephalus Patients presenting with concern for obstructive hydrocephalus underwent imaging demonstrating the presence of an obstructing contrast-enhancing mass. Patients for whom a gross total resection was deemed likely underwent tumor resection (upper row). ETV was the preferred method in patients who could not undergo gross total resection (middle row), with VPS reserved for patients who either failed prior ETV, for whom ETV was deemed technically unable to be performed, or for whom, clinically, it was thought unlikely to be therapeutic for the obstructing hydrocephalus (bottom row). Following each intervention, the patient was evaluated for resolution of hydrocephalus. ETV: endoscopic third ventriculostomy; VPS: ventriculoperitoneal shunt

Under an Institutional Review Board (IRB)-approved protocol and according to the Declaration of Helsinki, we performed a retrospective review of pediatric and adult patients with midline tumors over an eight-year period (September 14, 2016-July 1, 2024). All gliomas were classified into either a low (WHO I-II) or a high (WHO III-IV) grade according to the 2007 WHO Classification of Tumors of the Central Nervous System [9]. Inclusion criteria included pediatric and adult patients diagnosed with midline glioma by either brain MRI or pathologic confirmation. All patients underwent immunohistochemistry testing for the H3K27M mutant protein at Johns Hopkins Hospital or the Mayo Clinic. Hydrocephalus was confirmed on brain MRI. Patients without brain tumors who underwent ETV at our institution were used as a control. The determination of the specific CSF diversion procedure was based on several factors. If the lesion was resectable depending on the tumor’s location, a debulking procedure was preferred to restore normal CSF flow. Patients harboring tumors who were able to undergo a GTR were offered it. For unresectable lesions, an ETV was preferred over a VPS unless communicating hydrocephalus was present when a VPS was performed. VPS placement was performed using a standard neurosurgical technique. Utilizing stereotactic navigation, the neurosurgeon passed an antibiotic-impregnated ventricular drain via a cranial burr hole into the ventricle. This catheter was connected to an MRI-compatible, programmable valve with an anti-siphon device and connected to a distal peritoneal catheter placed laparoscopically by general surgery. ETV was performed using a rigid neuro-endoscope with a standard neurosurgical technique. Utilizing stereotactic navigation, an incision and burr hole were made for the optimal trajectory to fashion a third ventriculostomy. Endoscopic tumor biopsy was performed via the same burr hole when appropriate or via a separate burr hole when needed. Upon inspection of the third ventricle floor, a ventriculostomy was fashioned bluntly with forceps. The hole was expanded via a Fogerty-style balloon. The ventriculostomy was inspected to ensure that the underlying arachnoid was opened and that there were excellent CSF pulsations. The septum pellucidum would similarly be fenestrated as needed to release any entrapped ventricle. Figure 1 depicts a timeline of the management of a patient who presents with a midline brain tumor. Figure 2 highlights an algorithm for the treatment of obstructive hydrocephalus.

Data were analyzed to determine patient age and gender, WHO tumor grade (low versus high), extent of resection (biopsy, GTR, subtotal resection (STR)), hydrocephalus and CSF diversion procedures (tumor resection, ETV, VPS), presence of a H3K27 mutation, and overall survival (OS).

Statistical analysis

Patient characteristics were summarized using descriptive statistics. Chi-square tests and Fisher's exact tests were used to compare categorical variables, and nonparametric tests were used for continuous variables due to non-normal distributions. Each continuous variable was tested for normality using the Shapiro-Wilkes test. Age was reported as a mean (SD) since there was not enough evidence to reject the hypothesis of a normal distribution. For variables with significant p-values from the Shapiro-Wilkes test, indicating a non-normal distribution, the median (IQR) was reported, and the Wilcoxon Rank-Sum test was used for group comparisons. For normally distributed variables, such as age, the parametric t-test was used for group comparisons. Kaplan-Meier survival analyses were performed to assess time to hydrocephalus recurrence and overall survival (OS), with log-rank tests used for significance testing.

## Results

Clinical characteristics

A total of 74 patients were identified with midline gliomas (Table 1). The mean age was 33.2 years (range: 1.1-78.1 years), with 33 (44.6%) patients under the age of 18 years. The median age was lower in H3K27M patients (16.3 years) compared to WT (38.2 years), although this difference was not statistically significant (p=0.074). The majority (44 (59.5%)) of patients were female. Forty-eight (64.9%) patients had a high-grade midline glioma. Most (52 (70.3%)) patients underwent a biopsy while 7 (9.5%) had a GTR and 15 (20.3%) had a subtotal resection (STR).

**Table 1:** Clinical characteristics of patients who present with midline primary brain tumors * Fisher’s Exact; # Mann-Whitney; WT: wild type; H3K27M: histone 3 gene K27M mutation; ETV: endoscopic third ventriculostomy; VPS: ventriculoperitoneal shunt; OR: odds ratio; NA: not applicable

Characteristic	Overall (n=74)	Wild Type (n=54)	H3K27M Mutation (n=20)	Statistical Test	P-Value
Age (years)	23.8 (9.8, 60.5)	38.2 (11.7, 63.9)	16.3 (7.7, 33.5)	#, W=687	0.074
Group = Younger than 18 years	33 (44.6%)	22 (40.7%)	11 (55.0%)	*, OR=1.78 (0.63, 5.00)	0.303
Gender = Male	44 (59.5%)	32 (59.3%)	12 (60.0%)	*, OR=1.03 (0.36, 2.94)	1.000
Grade = Low grade	26 (35.1%)	25 (46.3%)	1 (5.0%)	*, OR=0.06 (0.01, 0.49)	0.001
Extent of Resection				*, OR=NA (NA, NA)	0.637
Biopsy	52 (70.3%)	36 (66.7%)	16 (80.0%)		
GTR	7 (9.5%)	6 (11.1%)	1 (5.0%)		
STR	15 (20.3%)	12 (22.2%)	3 (15.0%)		
Hydrocephalus	24 (32.4%)	9 (16.7%)	15 (75.0%)	*, OR=15.0 (4.34, 51.81)	<0.001
Hydrocephalus treatment				*, OR=NA (NA, NA)	0.027
ETV	5 (21.7%)	3 (37.5%)	2 (14.3%)		
ETV and VPS	4 (17.4%)	0 (0.0%)	4 (28.6%)		
Resection	5 (22.7%)	4 (50.0%)	1 (7.1%)		
VPS	8 (34.8%)	1 (12.5%)	7 (50.0%)		
VPS	12 (52.2%)	1 (11.1%)	11 (78.6%)	*, OR=29.3 (2.6, 336.4)	0.003
ETV	9 (39.1%)	3 (33.3%)	6 (42.9%)	*, OR=1.5 (0.26, 8.6)	1.000
Resection	5 (22.7%)	4 (50.0%)	1 (7.1%)	*, OR=0.077 (0.007, 0.901)	0.039
Duration between 1^st^ and 2^nd^ diversion procedures (days)	490.5 (45.0, 1104.5)	1140.5 (569.8, 1826.0)	143.5 (35.0, 494.8)	#, W=88.5	0.026
Alive at last follow-up = Yes	30 (40.5%)	26 (48.1%)	4 (20.0%)	*, OR=0.27 (0.08, 0.91)	0.035
Duration from midline glioma diagnosis to last follow-up (years)	1.5 (0.7, 5.0)	1.7 (0.7, 5.2)	1.3 (0.7, 2.2)	#, W=647	0.195
Duration between 1^st^ diversion procedure and last follow-up (months)	18.2 (7.2, 59.0)	53.6 (21.8, 70.2)	11.2 (6.8, 27.3)	#, W=82	0.076

Of the 74 patients, 20 (27.0%) were diagnosed with an H3K27M mutation and 54 (73.0%) were WT. Patients with a WT midline glioma were statistically more likely to have a low-grade midline glioma compared to those with a DMG H3K27M mutation (p < 0.001). There was no statistical difference in age, gender, or extent of resection between patients with WT or H3K27M gliomas. 

Hydrocephalus 

Twenty-four patients (32.4%) patients developed hydrocephalus, including 15 (75.0%) with a DMG H3K27M mutation and 9 (16.7%) with a WT midline glioma. Of the 24 patients with hydrocephalus, 18 (75%) were diagnosed with the midline glioma concurrently with the hydrocephalus. Of the 6 (25%) patients whose midline glioma was diagnosed before the hydrocephalus, the mean duration between the diagnosis of the midline glioma and the diagnosis of the hydrocephalus was 10.57 months (1.6-20.23 months).

Patients with a DMG H3K27M mutation were statistically more likely to be diagnosed with hydrocephalus versus those with a WT midline glioma (p<0.001). Of the total 24 patients with hydrocephalus, 8 (34.8%) underwent a VPS placement, 5 (22.7%) had a tumor resection, 5 (21.7%) underwent an ETV, and 4 (17.4%) had both a VPS and ETV. There was a statistically significant difference among these treatments between the H3K27M and WT midline glioma groups (p=0.027). Two patients did not undergo a procedure for the hydrocephalus. Of the 12 (52.2%) patients who had a VPS shunt inserted, 11 (78.6%) had a DMG H3K27M mutation compared to 1 (11.1%) with a WT midline glioma (p=0.003). Of the 9 (39.1%) patients who had an ETV procedure, 6 (42.9%) had a DMG H3K27M mutation versus 3 (33.3%) with a WT midline glioma (p=1.000). Of the 5 (22.7%) patients who were treated with tumor resection, 4 had a WT midline glioma compared with 1 with DMG H3K27M mutation (p=0.039).

**Figure 3:**
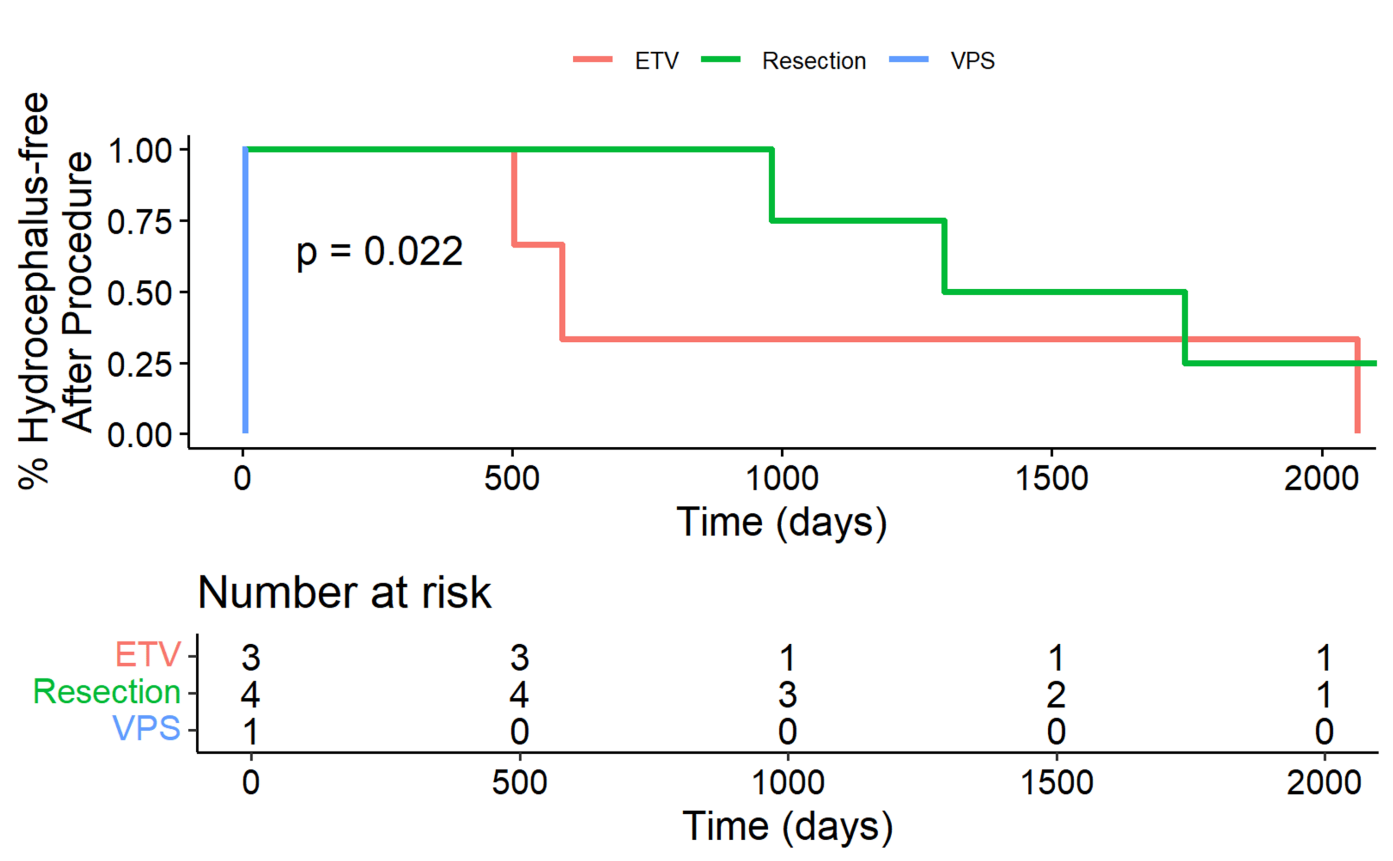
Kaplan-Meier plot of wild-type patients who remained free of hydrocephalus This Kaplan-Meier plot shows the percentage of wild-type (WT) patients who remained free of hydrocephalus over time, stratified by treatment type: endoscopic third ventriculostomy (ETV), tumor resection, and ventriculoperitoneal shunt (VPS). The x-axis represents time in years, and the y-axis represents the percentage of patients without hydrocephalus. The number-at-risk table below the plot displays the number of patients remaining in each group at different time intervals. The log-rank test p-value of 0.022 indicates a significant difference in hydrocephalus-free survival among the treatment groups.

The patients with a WT midline glioma who remained free of hydrocephalus over time after the initial diversion procedure are presented in Figure 3, stratified by treatment type (ETV, tumor resection, and VPS). A significant difference in hydrocephalus-free survival was noted among the treatment groups (p=0.022). Patients who underwent tumor resection exhibited the longest hydrocephalus-free survival times. The Kaplan-Meier curve for the resection group showed the first decline in survival probability around day 1000, gradually decreasing to approximately 25% by day 2000 (Figure 3). In contrast, patients who underwent ETV experienced a decrease in hydrocephalus-free survival starting around day 300, with the probability declining to about 33% by day 2000. The single patient who underwent a VPS placement experienced a rapid decline, with hydrocephalus occurring around day 150.

**Figure 4:**
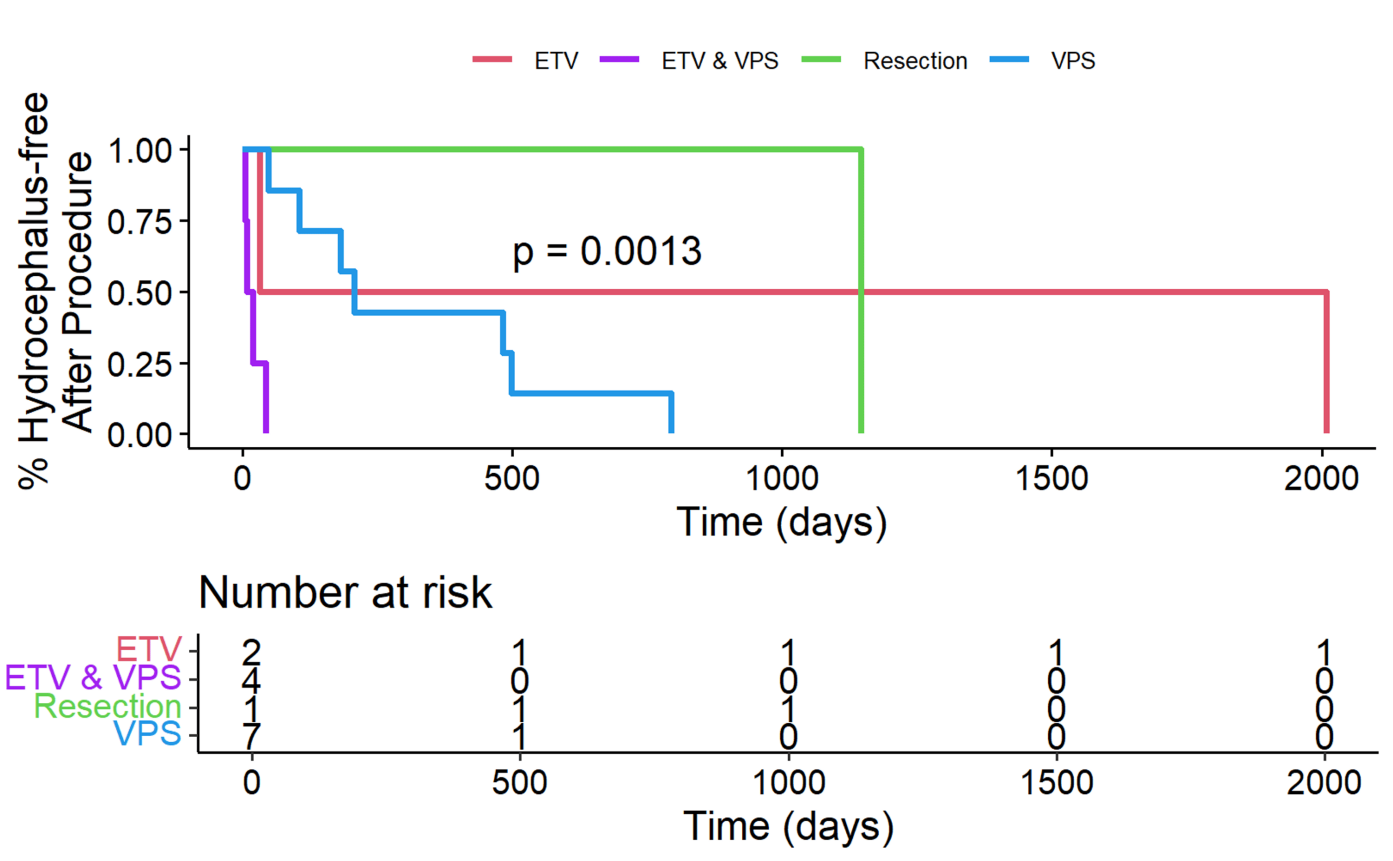
Kaplan-Meier plot of patients with an H3K27M mutation who remained free of hydrocephalus This Kaplan-Meier plot illustrates the percentage of patients with the H3K27M mutation who remained free of hydrocephalus over time, stratified by treatment type: ETV, ETV and VPS, tumor resection, and VPS. The x-axis represents time in days, and the y-axis represents the percentage of patients without hydrocephalus. The number-at-risk table below the plot shows the number of patients remaining in each group at various time points. The log-rank test p-value of 0.0013 indicates a significant difference in hydrocephalus-free survival among the treatment groups. ETV: endoscopic third ventriculostomy; VPS: ventriculoperitoneal shunt

Patients with a DMG H3K27M mutation who remained free of hydrocephalus over time after the first diversion procedure are shown in Figure 4, stratified by ETV, ETV/VPS, tumor resection, and VPS. A significant difference in hydrocephalus-free survival was observed among the treatment groups (p=0.0013). ETV failure was significantly higher in patients with a DMG H3K27M mutation while VPS was more successful in managing hydrocephalus in this group.

Four patients underwent two diversion procedures for hydrocephalus, three of whom initially had an ETV followed by a VPS and the fourth underwent tumor resection on two occasions. One patient underwent three diversion procedures, with two ETVs followed by a VPS. One patient had four diversion procedures, starting with one VPS, then two ETVs, and finally another VPS. Five of these six patients had a DMG H3K27M mutation. The duration between the first and second diversion procedure was significantly longer in the WT midline glioma group: 1140.5 (569.8, 1826.0) days, as compared to the DMG H3K27M mutation group: 143.5 (35.0, 494.8) days (p=0.026).

Overall survival

At last follow-up, 30 (40.5%) patients were still alive. A statistically higher number of patients (26 (48.1%)) with a WT midline glioma were still alive compared to those with a DMG H3K27M mutation (4 (20.0%)) (p=0.035). The median duration between midline glioma diagnosis and last follow-up for all patients was 1.5 years (0.7, 5.0), with no statistically significant difference between H3K27M (1.3 (0.7, 2.2)) and WT (1.7 (0.7, 5.2)) groups (p=0.195).

**Figure 5:**
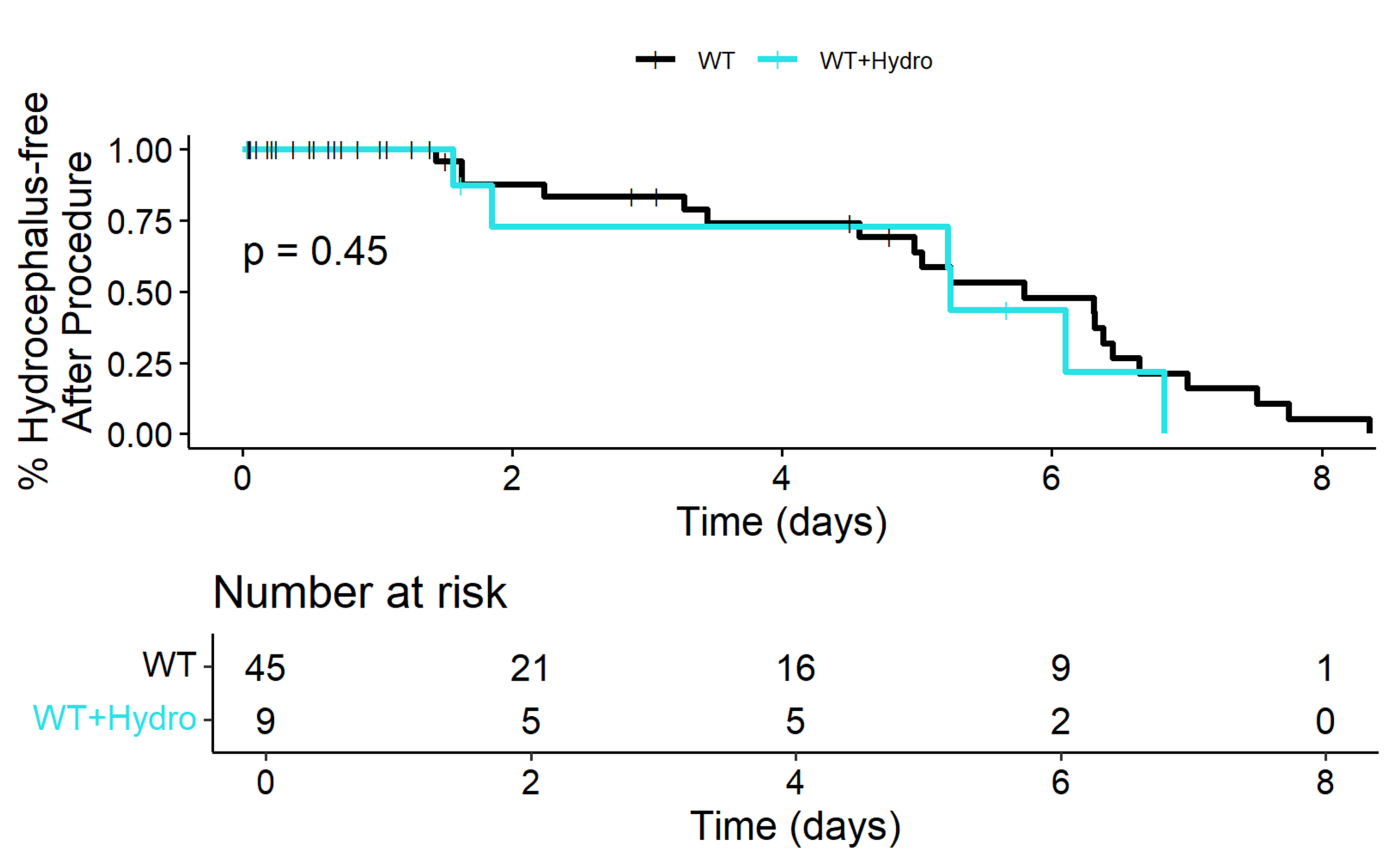
Kaplan-Meier plot of overall survival of patients with wild-type midline gliomas with and without hydrocephalus This Kaplan-Meier plot compares the overall survival of patients with wild-type (WT) midline gliomas with and without hydrocephalus. The x-axis represents the time in years, and the y-axis represents the overall survival probability. The number-at-risk table indicates the number of patients remaining in each group at different time intervals. The log-rank test p-value of 0.45 suggests no significant difference in overall survival between WT patients with and without hydrocephalus.

**Figure 6:**
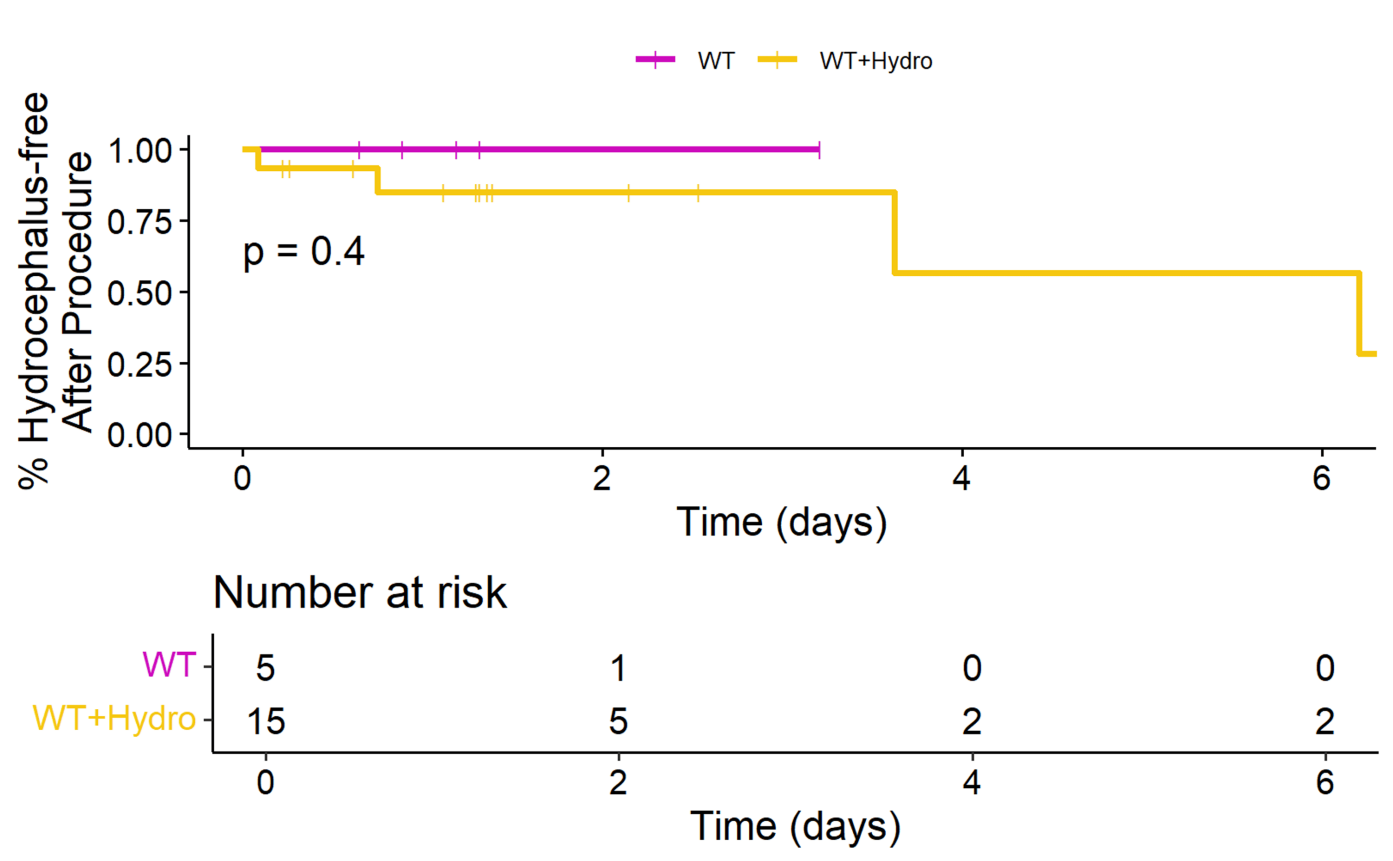
Kaplan-Meier plot of overall survival of patients with H3K27M mutation with and without hydrocephalus This Kaplan-Meier plot compares the overall survival of patients with the H3K27M mutation with and without hydrocephalus. The x-axis represents the time in years, and the y-axis represents the overall survival probability. The number-at-risk table below the plot shows the number of patients remaining in each group at various time points. The log-rank test p-value of 0.40 indicates no significant difference in overall survival between H3K27M patients with and without hydrocephalus.

No significant difference was observed in OS between WT patients with and without hydrocephalus (p=0.45) (Figure 5). Similarly, no significant difference was noted in OS between patients with a DMG H3K27M mutation with and without hydrocephalus (p=0.4) (Figure 6).

Pediatric patients versus adults with a DMG H3K27M mutation

The clinical characteristics of patients with a H3K27M mutation, comparing adults (> 18 years) to pediatric patients (≤ 18 years), are summarized in Table 2. Of the 20 patients with a DMG H3K27M mutation, 11 (55%) were younger than age 18 while 9 (45%) were older than 18. There was a statistically significant difference in age between the two groups: the median age of the adults was 37.2 years as compared to 8.3 years in the pediatric group (p <0.001). No significant differences were noted in gender, tumor grade, extent of resection, hydrocephalus incidence, OS, or time-related variables between adults and children. 

**Table 2:** Clinical characteristics of pediatric versus adult patients with diffuse midline glioma H3K27M mutation * Fisher’s Exact; # Mann-Whitney; WT: wild type; H3K27M: histone 3 gene K27M mutation; ETV: endoscopic third ventriculostomy; VPS: ventriculoperitoneal shunt; OR: odds ratio; NA: not applicable

Characteristic	Overall (n=20)	Adults >18 years (n=9)	Pediatrics ≤ 18 years (n=11)	Statistical test	P-Value
Age (years)	16.3 (7.7, 33.5)	37.2 (27.0, 60.2)	8.3 (5.5, 11.9)	#, W=99	< 0.001
Gender = Male	12 (60.0%)	7 (77.8%)	5 (45.5%)	*, OR=0.24 (0.03, 1.71)	0.197
Grade = Low Grade	1 (5.0%)	1 (11.1%)	0 (0.0%)	*, OR=NA (NA, NA)	0.450
Extent of Resection				*, OR=NA (NA, NA)	0.770
Biopsy	16 (80.0%)	7 (77.8%)	9 (81.8%)		
GTR	1 (5.0%)	1 (11.1%)	0 (0.0%)		
STR	3 (15.0%)	1 (11.1%)	2 (18.2%)		
Hydrocephalus	15 (75.0%)	8 (88.9%)	7 (63.6%)	*, OR=0.22 (0.02, 2.45)	0.319
Hydrocephalus treatment				*, OR=NA (NA, NA)	0.212
ETV	2 (14.3%)	2 (25.0%)	0 (0.0%)		
ETV and VPS	4 (28.6%)	3 (37.5%)	1 (16.7%)		
Resection	1 (7.1%)	1 (12.5%)	0 (0.0%)		
VPS	7 (50.0%)	2 (25.0%)	5 (83.3%)		
VPS	11 (78.6%)	5 (62.5%)	6 (100.0%)	*, OR=NA (NA, NA)	0.209
ETV	6 (42.9%)	5 (62.5%)	1 (16.7%)	*, OR=0.12 (0.009, 1.58)	0.138
Resection	1 (7.1%)	1 (12.5%)	0 (0.0%)	*, OR=NA (NA, NA)	1.000
Alive at last follow-up = Yes	4 (20.0%)	3 (33.3%)	1 (9.1%)	*, OR=0.22 (0.02, 2.45)	0.285
Duration from midline glioma diagnosis to last follow-up (years)	1.3 (0.7, 2.2)	1.1 (0.6, 3.6)	1.3 (1.1, 1.4)	#, W=49	0.970
Duration between 1^st^ diversion procedure and last follow-up (months)	11.2 (6.8, 27.3)	22.0 (6.0, 51.2)	8.1 (6.8, 14.3)	#, W=31	0.366

## Discussion

Hydrocephalus in the setting of a DMG H3K27M mutation has rarely been reported in the literature [1,18-24]. It may develop by either mass effect on the foramen of Monroe, Sylvian aqueduct, or fourth ventricle causing CSF outflow obstruction or by disseminated leptomeningeal disease leading to reduced CSF reabsorption [20,25]. Less than 10% of patients with DIPG have dorsal extension of the tumor that blocks CSF flow leading to hydrocephalus [13]. However, leptomeningeal dissemination in DIPG may occur in up to 50% of patients [22,25]. Temporary CSF diversion techniques comprise lumbar drains, external ventricular drains, and Ommaya reservoirs [20]. Two surgical procedures are commonly utilized to treat hydrocephalus, including a VP shunt where CSF is diverted from the cerebral ventricles into the peritoneal cavity and an ETV involving fenestration in the third ventricular floor permitting CSF to flow to the subarachnoid cistern anterior to the brainstem to bypass the obstructed fourth ventricle [20]. While a VP shunt is a more frequent method, it is associated with numerous risk factors including infection, blockage of the shunt which may necessitate a revision, or tumor dissemination through the shunt tubing into the abdominal cavity [20,22]. ETV is a minimally invasive procedure, does not have a permanent implant, and is associated with high rates of success [20,25].

In Baugh and colleagues’ study of 582 patients with DIPG, 86 (14%) had evidence of hydrocephalus at the time of the DIPG diagnosis [19]. The median OS of the 43 patients with hydrocephalus who were treated with a CSF diversion procedure was 13 months compared to 9 months for the 43 patients with hydrocephalus who did not undergo CSF diversion. The survival rates between these two groups were not significantly different. However, patients with hydrocephalus who had cranial nerve palsy at diagnosis and subsequently underwent a CSF diversion procedure had a significantly reduced risk. In Fonseca and colleagues’ study of 82 patients with DIPG, 8 patients had symptomatic hydrocephalus and underwent a CSF diversion procedure (VPS placed in 6) [21]. At tumor progression, 36 (55%) patients had evidence of ventriculomegaly, 9 of whom had a CSF diversion procedure. The authors concluded that CSF diversion for hydrocephalus at the time of diagnosis does not impact survival; however, patients with symptomatic hydrocephalus at progression and underwent CSF diversion had a survival advantage compared to patients with ventriculomegaly who were treated conservatively [21]. Three complications were associated with the VPS, including (1) an inguinal hernia containing the VPS tubing that required surgical repair, (2) a shunt infection that necessitated externalization of the drain, and (3) nodular lesions along the path of the VPS tubing that were dissemination of the DMG H3K27M.

In Amano and colleagues’ study of 18 patients under the age of 15 years with a brainstem glioma, 16 (88.9%) patients were diagnosed with hydrocephalus [18]. The average time to hydrocephalus after tumor diagnosis was 5.1 months. Twelve of the 16 patients with hydrocephalus were treated with a VPS shunt, ETV, or Torkildsen shunt. Patients treated for hydrocephalus survived significantly longer than those who did not undergo hydrocephalus management. These authors reported that CSF diversion significantly improves the quality of life and survival of patients [18]. In Roujeau and colleagues’ study of 51 children with brainstem gliomas, 11 (22%) patients developed hydrocephalus, 9 of whom had a VPS and 2 of whom had an ETV [24]. The median duration from tumor diagnosis to hydrocephalus onset was 3.2 months and from hydrocephalus onset to death was 2.8 months. The survival rate of patients with obstructive hydrocephalus was not significantly different from those who did not develop hydrocephalus. These authors opined that VPS should be the leading diversion technique to treat hydrocephalus [24].

In Guida and colleagues’ systematic review of 55 patients (6 studies) with DIPG-related hydrocephalus and treated with either ETV (37 (67%)), VPS (16 (29%)), or VPS according to Torkildsen (2 (4%)), 35 (86%) patients who underwent an ETV had clinical improvement after surgery with only minor bleeding that resolved with continuous irrigation during surgery [25]. The mean time of hydrocephalus onset from tumor diagnosis was reported in 2 studies and was 5.25 months. VPS placement was associated with a 50% failure rate. Two patients with a VPS had a shunt malfunction, and 10% had increased ventricular sizes. There was no statistical difference between the efficacy of VPS and ETV.

Single case reports have also been reported where a patient experienced hydrocephalus concurrent with the diffuse midline glioma with an H3K27M mutation [1,22,26,27]. Chen et al. described a case where the DMG was located in the prepontine cistern, and the patients had diffuse involvement of the meninges and communicating hydrocephalus [26]. A VPS was inserted, and the patient’s headaches resolved. In Yekula et al.’s case report of a patient with DMG H3K27M with gliomatosis cerebri growth (diffuse, bilateral involvement of multiple deep structures with extension into supra- and infratentorial white matter and leptomeninges), communicating hydrocephalus and papilloma were noted [27]. One month following VPS insertion, the visual symptoms recurred, requiring a VPS revision. Additional shunt failures and revisions occurred. Gelder et al. reported a case of subcutaneous spread of DIPG along the VPS track [22].

In our work, ETV had a high failure rate in patients who were subsequently determined to harbor the DMG H3K27M mutation. For patients with a WT midline glioma, tumor resection to treat hydrocephalus offered the longest hydrocephalus-free survival times. Contrasting with previous studies in the literature where the tumor was diagnosed between 3.2-5.25 months before the hydrocephalus, most (75%) patients in our study were diagnosed with midline glioma and hydrocephalus concurrently. Of the 6 (25%) patients whose midline glioma was diagnosed before the hydrocephalus, the mean duration was 10.57 months. As a control, of the 100 patients without brain tumors who underwent ETV at our institution, 84 had patency at the last follow-up. By controlling for procedure-specific factors, we were able to effectively analyze tumor-specific factors between the patients with and without the H3K27M mutation. We postulate that the H3K27M mutation conveys an overall inflammatory response, which causes scarring of the fenestrated ventriculostomy, resulting in early failure. Additional studies of the ventricular lining would be necessary to test this hypothesis and may be a future focus for our studies. The correlation of early ETV failure was only significant in the H3K27 mutation and tumor group. We postulate that this is due to an inflammatory response to the mechanical injury of fenestration. Of the 15 patients with hydrocephalus who subsequently were found to have H3K27M mutations, 11 had CSF sampling done. Only 2 of these 11 had tumor cells present on CSF cytology. Therefore, it would seem that another mechanism besides leptomeningeal carcinomatosis underlies the ETV failure. The prevalence of the H3K27M mutation is histology and age-dependent. This work tested the hypothesis that regardless of age, patients who fail an ETV should have their tumor assayed for the presence of the H3K27M mutation. Our initial study of hydrocephalus has focused on survival and hydrocephalus resolution. As the quality of life and activities of daily living also matter in this patient population, we plan to collect these data in our next analysis to determine if hydrocephalus resolution improves these endpoints.

In addition to the clinical, radiological, and histological findings, immunohistochemistry for the H3K27M mutation of all midline gliomas is important regardless of patient age or histological appearance to permit an accurate risk stratification, prognosis, and treatment guidance [3,5,11,15,28]. In Meyronet and colleagues’ study of 21 adults with a DMG H3K27M mutation compared to 135 adult diffuse gliomas without the mutation, 1 in 5 patients had a histopathological presentation that did not suggest a high-grade diffuse glioma [7]. These authors reported that the mutation identification had a significant impact on the diagnosis [7].

Strengths and limitations of the current study

 The strength of the present study is that we evaluated pediatric and adult patients with midline gliomas who were diagnosed with an H3K27M mutation, developed hydrocephalus, and underwent tumor resection or a CSF diversion procedure. This study adds to the literature about the safety and efficacy of VPS and ETV in treating patients with a DMG H3K27M mutation and hydrocephalus. Additionally, our findings of the superior outcomes of VPS compared to ETV may aid neurosurgeons in their decision-making process when evaluating patients with a midline glioma with hydrocephalus. As molecular testing often takes up to two weeks to be reported, it is important to effectively manage patients with a midline brain tumor when they present to the Emergency Department if they have evidence of hydrocephalus (Figure 1). If patients with a midline brain tumor undergo an ETV and fail, these patients most likely harbor the H3K27M mutation which has oncologic consequences. The main point of this paper is that when a patient without a pathologic diagnosis fails an ETV, the neurosurgeon and neuro-oncology team should consider that patient to harbor a tumor with an H3K27 mutation. The single patient who underwent a VPS placement and experienced a rapid decline with hydrocephalus occurring around day 150 had an underlying WT tumor. Statistically, this reduces the power when compared within the WT group to make determinations regarding the use of VPS. However, we included this patient to allow the reader to compare the datasets between WT (Figure 3) and K27MH3 (Figure 4) patients. The case in Figure 3 was treated with an initial VPS and died on postoperative day 6 from a pulmonary embolus without detectable hydrocephalus. Thus, this patient did not appear to have shunt obstruction. In Figure 4, the four patients with ETV with subsequent VPS were all found to have a diffuse midline glioma with H3K27M mutation as the underlying tumor histology. The underlying mechanism for the relatively rapid ETV failure remains undetermined.

The limitations of the current study are its small number of patients and its retrospective nature. The study subgrouping reduces the statistical power to determine which method would be most appropriate prospectively. The main attempt of this work was to show that regardless of the management decisions for obstructive hydrocephalus, clinicians should monitor ETV patients for early failure unless histology and H3K27M mutant status are known. Given the limitation regarding the statistical power of the log-rank test due to the small number of non-affected subjects, alternative survival modeling approaches, such as the Cox proportional hazards model, would likely face similar challenges related to small sample sizes and sparse events, which could affect the stability of estimates. While the log-rank test remains an appropriate choice for group comparisons, we acknowledge its limitations and have ensured that interpretations reflect the sample size constraints. In future work, approaches such as restricted mean survival time analysis or Bayesian survival models could be considered to address these challenges.

## Conclusions

DMGs are a subset of malignant gliomas with an H3K27M mutation. Hydrocephalus may be the presenting condition. The outcomes of tumor resection, ETV, and VPS placement in DMG patients compared to WT tumors in treating hydrocephalus were assessed. The H3K27M mutation is associated with younger age, higher hydrocephalus incidence, and shorter duration from the initial hydrocephalus diversion procedure to the last follow-up. The high prevalence of hydrocephalus in patients with a DMG H3K27M mutation underscores the need for effective management strategies. VPS demonstrated superior outcomes as compared to ETV in controlling hydrocephalus among patients with a DMG H3K27M mutation. Despite the high failure rate of ETV in this group, OS was not significantly impacted by hydrocephalus. Neurosurgeons should have a high index of suspicion for ETV failure in the DMG-mutated patient population.
